# GLP-1 receptor agonists: exploration of transformation from metabolic regulation to multi-organ therapy

**DOI:** 10.3389/fphar.2025.1675552

**Published:** 2025-09-11

**Authors:** Bing Gong, Couwen Li, Zhuang’e Shi, FuPing Wang, Ruanxian Dai, Guobing Chen, Heng Su

**Affiliations:** ^1^ Faculty of Life Science and Technology, Kunming University of Science and Technology, Kunming, China; ^2^ Medical School, Kunming University of Science and Technology, Kunming, China; ^3^ Department of Emergency Medicine, The First People’s Hospital of Yunnan Province, Kunming, China; ^4^ The Affiliated Hospital of Kunming University of Science and Technology, Kunming, Yunnan, China

**Keywords:** GLP-1 receptor agonist, molecular mechanisms, gut microbiota, multi-organ protection, therapeutic repurposing

## Abstract

Glucagon-like peptide-1 receptor agonists (GLP-1RAs), initially developed for type 2 diabetes and obesity, have evolved into multi-organ potential therapeutics due to their pleiotropic effects beyond glycemic control. Mechanistically, GLP-1 signaling modulates immune and inflammatory pathways, regulates autophagy and pyroptosis, alleviates endoplasmic reticulum stress, and interacts with the gut microbiome. These pleiotropic effects provide a rationale for exploring their role in multiple organ systems. Clinical trials have demonstrated cardiovascular and renal protection, leading to additional approvals in high-risk populations. Early data also suggest potential benefits in liver disease, obstructive sleep apnea, chronic respiratory disorders, neurodegenerative and psychiatric conditions, reproductive dysfunction, obesity-associated cancers, and sepsis, although these remain investigational. Therefore, this review aims to synthesize the evidence on the mechanistic expansion of GLP-1RAs from metabolic regulators to systemic modulators of inflammation, autophagy, and organ protection, and explores their therapeutic repurposing across diseases.

## 1 Introduction

Glucagon-like peptide-1 (GLP-1) belongs to a class of intestinally derived hormones known as incretins (Bany Bakar, 2023). It originates from the post-translational modification of the proglucagon precursor and exerts diverse metabolic functions via binding to the GLP-1 receptor (GLP-1R), a class B G protein-coupled receptor (GPCR) that is extensively distributed among numerous organ systems (Aroda, 2018).

Initial studies identified the pancreas as the primary site of GLP-1R expression. However, with the progression of research, it has become evident that GLP-1R is broadly distributed across various organs and tissues, including but not limited to the brain, heart, kidneys, intestines, eyes, and the vascular system (Femminella et al., 2019; Meier, 2012; Rakipovski et al., 2018). Moreover, GLP-1R expression has also been detected in several components of the immune system, such as macrophages, lymphocytes, and invariant natural killer T (iNKT) cells (Song et al., 2017). This wide-ranging receptor localization emphasizes GLP-1’s diverse physiological impacts and supports its role as a potential therapeutic agent in treating a variety of disorders, including metabolic dysfunctions, cardiovascular diseases, and chronic inflammatory conditions.

GLP-1 has gained prominence not only for its receptor distribution across multiple systems, but also for its broad metabolic regulatory activities. These include facilitating glucose reduction, delaying gastric motility, improving insulin responsiveness, and inhibiting glucagon production (Meier, 2012). One of its key physiological roles involves the modulation of postprandial glycemic fluctuations, primarily achieved through stimulating insulin release and concurrently diminishing glucagon output. Since the discovery and functional verification of GLP-1 in the 1980s (Kreymann et al., 1987), it has markedly reshaped current clinical approaches to the treatment of obesity and type 2 diabetes, providing superior glycemic control with reduced hypoglycemic risks compared to conventional therapies.

However, the therapeutic utility of native GLP-1 was limited by its rapid enzymatic degradation and short plasma half-life, which prompted the development of longer-acting receptor agonists. Exendin-4, identified in 1992 from lizard venom, shares approximately 53% sequence similarity with human GLP-1. This peptide is notable for its resistance to degradation by dipeptidyl peptidase-4 (DPP-4) and its relatively slow renal clearance. GLP-1 receptor agonists (GLP-1RAs) were designed to reproduce endogenous GLP-1 biology while overcoming the peptide’s pharmacokinetic liabilities.

Exenatide, the first GLP-1RA approved in 2005, demonstrated clinical efficacy but its low homology with human GLP-1 and gastrointestinal side effects restricted widespread use (Muttenthaler et al., 2021). In 2009, liraglutide—a modified human GLP-1 analogue featuring an extended duration of action via fatty acid conjugation and exhibiting approximately 97% sequence identity with native GLP-1—was first authorized for therapeutic use in Europe. Since then, the evolution of GLP-1 receptor agonists has progressed from initial short-duration agents like exenatide, to intermediate-duration formulations such as liraglutide, and further to advanced long-acting options including dulaglutide and semaglutide (The two products received approval for marketing in the U.S. in 2014 and 2017, respectively) (Pfeffer et al., 2015).

More recently, multi-agonist incretin therapies have expanded the GLP-1 paradigm toward greater weight and metabolic benefits. In 2022, tirzepatide was launched, becoming the world’s first new-generation drug that acts on both GLP-1R/GIPR targets. In addition to regulating blood sugar and lowering body weight in non-diabetic obese patients, tirzepatide can also lower liver fat and improve kidney outcomes (Heerspink et al., 2022). At the same time, triple receptor agonists are also rapidly developing. In 2023, retatrutide—a triagonist targeting GLP-1, GIP, and glucagon receptors demonstrated favorable efficacy outcomes in a phase II randomized, double-blind clinical study. This agent exhibited the potential to induce significant body weight reduction alongside improvements in cardiometabolic health parameters, potentially becoming the most potent weight-loss and anti-diabetic therapy in the field of metabolic diseases (Jastreboff et al., 2023). In addition, Oral, non-peptide GLP-1RA development may further change the treatment landscape by removing injection-related barriers. In 2025, Orforglipron, the first non-peptide oral GLP-1 receptor agonist, successfully completed Phase III trials. If approved, it could overcome the current limitations in drug delivery and transform the treatment landscape (Rosenstock et al., 2025).

Beyond their role in glucose metabolism, GLP-1 and its receptor agonists have emerged as multifunctional agents with broad cardiometabolic, renal, and immunomodulatory benefits. Clinical data further reinforce these mechanistic insights. For example, the SELECT trial clearly demonstrated that semaglutide significantly reduces the risk of major adverse cardiovascular events (MACE), leading to FDA approval for lowering the risk of cardiovascular death, myocardial infarction, or stroke in overweight or obese adults with established cardiovascular disease (Lincoff et al., 2023; Marso et al., 2016a). In 2024, tirzepatide also gained authorization for managing moderate-to-severe obstructive sleep apnea (OSA) in obese adults (Malhotra et al., 2024). Furthermore, in January 2025, the FDA approved semaglutide (Ozempic^®^) for reducing the risk of kidney failure and disease progression in patients with diabetes and chronic kidney disease (CKD), making it the first GLP-1 agent indicated for the treatment of CKD (Perkovic et al., 2024).

**FIGURE 1:**
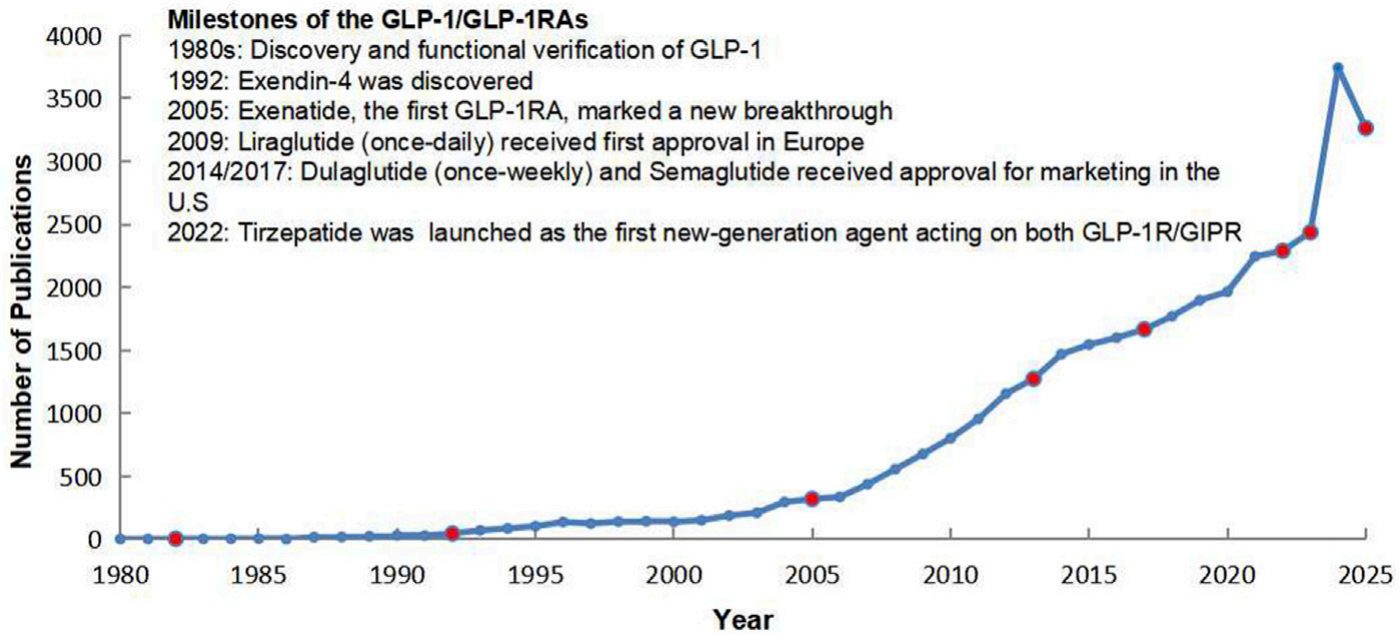
Milestones and publication trends of GLP-1/GLP-1 receptor agonists (1980–2025).

In parallel with these clinical advances, the growth of academic output has been striking. Publication numbers remained modest in the early decades but began to rise after the introduction of exenatide and liraglutide. Since 2015, with the approval of once-weekly formulations and the emergence of multi-target agents, the field has witnessed exponential growth in publications. By 2025, the cumulative number of scientific articles on GLP-1 and GLP-1RAs had increased more than tenfold compared with 2005, reflecting the expanding scientific and clinical interest in this therapeutic area (Figure 1).

Taken together, these scientific milestones and the rapid rise in scholarly output highlight the momentum of GLP-1–based therapies. They underscore not only the clinical impact of this drug class but also the urgent need to comprehensively review the evolving evidence base, clarify mechanistic insights, and explore future directions in both metabolic and non-metabolic disease contexts.

## 2 Molecular and cellular mechanisms of GLP-1

### 2.1 Metabolic regulation of glucose homeostasis

The classical pathophysiological mechanisms of GLP-1 have been well studied. GLP-1 activates the GLP-1R to stimulate adenylate cyclase (AC), increasing intracellular levels of cyclic AMP (cAMP) (Campbell and Drucker, 2013; Sandoval and D’Alessio, 2015). cAMP signaling stimulates PKA and EPAC, both of which contribute to the production and release of insulin (Yang and Yang, 2016). Activation of GLP-1R also triggers the PI3K/Akt signaling pathway via the βγ subunits of G proteins, thereby promoting β-cell viability and replicative capacity (Kaihara et al., 2013). However, emerging evidence suggests that the actions of GLP-1 are not limited to glycemic control.

### 2.2 Inflammation and immune modulation

Recent preclinical studies have demonstrated its bidirectional immunomodulatory effects, including improvement of organ dysfunction, regulation of immune responses, and mitigation of sepsis-associated cytokine storms, mediated through anti-inflammatory and anti-apoptotic pathways. Interestingly, certain classical inflammatory stimuli—including lipopolysaccharide (LPS) and pro-inflammatory cytokines such as interleukin-6 (IL-6) and tumor necrosis factor-α (TNF-α) can, in turn, induce GLP-1 secretion. These findings underscore GLP-1 as a pivotal molecular link bridging metabolic and immune pathways (Leonardini et al., 2017; Lincoff et al., 2023).

When intestinal lipopolysaccharide (LPS) causes metabolic syndrome, the individual’s microbiota metabolites and AhR activity are impaired. Activation of AhR stimulates GLP-1 secretion from intestinal enteroendocrine cells (EECs), and this effect is amplified by GLP-1RAs such as exendin-4. These agonists not only enhance GLP-1 release but also suppress the expression of pro-inflammatory cytokines, including IL-2, IL-17a, interferon-γ, and TNF-α (Yusta et al., 2015). Liraglutide suppresses TNF-α and monocyte chemoattractant protein-1 (MCP-1) expression in monocytes derived from healthy participants. In a mouse model of acute inflammation *in vivo*, with the treatment of sommarutide, circulating concentrations of pro-inflammatory cytokines, including TNF-α and IFN-γ, were decreased, accompanied by attenuated infiltration of immune effector cells (Hammoud and Drucker, 2023).

#### 2.2.1 Autophagy

Autophagy is an evolutionarily conserved catabolic process essential for cellular homeostasis during stress conditions, including nutrient deprivation and hypoxia. This lysosomal degradation pathway facilitates the recycling of damaged cellular components to maintain metabolic equilibrium. Its role exhibits context-dependent duality, functioning as a cytoprotective mechanism or contributing to regulated cell death (Levine and Kroemer, 2019). The PI3K/Akt/mTOR signaling axis serves as a primary regulator of the process. GLP-1RAs, particularly liraglutide, can modulate this pathway in disease-specific contexts: In neonatal hypoxic-ischemic brain injury models, liraglutide activates PI3K/Akt signaling, reducing neuronal apoptosis and improving long-term cognitive outcomes (Zeng et al., 2019; Yao et al., 2021). Similarly, Yao et al. demonstrated protective effects on nucleus pulposus cells under glucose toxicity through modulation of the same pathway. Beyond the nervous system, liraglutide intervention alleviates doxorubicin-induced testicular damage and cognitive dysfunction by counteracting oxidative injury and restoring PI3K/Akt/mTOR signaling homeostasis. In the vascular system, oxidative stress in diabetes activates ROS-mediated Akt and extracellular signal-regulated kinase 1/2 (ERK1/2) signaling cascades, promoting autophagy in endothelial cells while downregulating HDAC6 expression, which impairs cell migration and adhesion. GLP-1, however, engages the GLP-1R/ERK1/2/HDAC6 axis to modulate downstream transcriptional activity, it lowers intracellular ROS, reduces Akt and ERK1/2 phosphorylation, restores HDAC6 expression, and thereby alleviates endothelial dysfunction and excessive autophagy (Cai et al., 2018). At the pancreatic level, glucolipotoxicity in β-cells leads to accumulation of autophagy marker LC3 II, impaired lysosomal function, and blocked autophagic flux, ultimately driving cell death. Exendin-4 treatment rescued lysosomal activity, normalized autophagic clearance, and enhanced autophagic flux, thus preserving β-cell viability (Zummo et al., 2017).

#### 2.2.2 Pyroptosis

Activation of the NLRP3 inflammasome is a critical trigger of pyroptosis, leading to gasdermin-mediated membrane pore formation and excessive release of pro-inflammatory cytokines such as IL-1β and IL-18. GLP-1RAs, particularly Liraglutide exert protective effects by suppressing inflammasome activation and downstream pyroptotic responses.In H9c2 cells. It mitigated pyroptotic responses through modulation of the SIRT1/NOX4/ROS axis, thereby preventing gasdermin-dependent pore formation and cytokine overproduction (Chen et al., 2018). Subsequent studies further support its anti-pyroptotic effects across multiple cell types. For instance, in both H9c2 cardiomyoblasts and HepG2 hepatocellular carcinoma cells, liraglutide reduces pyroptosis by upregulating SIRT1 expression, scavenging intracellular reactive oxygen species (ROS), and enhancing mitophagy (Yu et al., 2019). Beyond liraglutide, other GLP-1RAs demonstrate similar properties. In db/db mice, exendin-4 (EX-4) significantly suppressed the overexpression of pyroptosis-related proteins—including ASC, caspase-1, IL-1β, and GSDMD. These findings indicate that GLP-1R activation specifically inhibits GSDMD-mediated microglial pyroptosis, a finding consistently replicated in subsequent *in vitro* experiments (Song et al., 2022).

#### 2.2.3 Macrophage polarization

Macrophage polarization represents a pivotal mechanism in shaping immune responses. Macrophages can polarize into two distinct phenotypes: M1, which exhibits pro-inflammatory properties, and M2, which is associated with anti-inflammatory functions (Zhang et al., 2023; Yao et al., 2019). Mechanistically, GLP-1 signaling suppresses M1-associated genes while enhancing that of M2-related genes through activation of signal transducer and activator of transcription 3 (STAT3) (Wan and Sun, 2019). In models of nonalcoholic steatohepatitis (NASH), GLP-1 analogs have been shown to attenuate hepatic inflammation by reducing M1 macrophage infiltration and inhibiting NF-κB activity, thereby lowering the expression of pro-inflammatory mediators such as IL-6, TNF-α, and iNOS (Yusta et al., 2015). *In vitro*, liraglutide dose-dependently downregulates pro-inflammatory mediators such as Mcp-1 and Cd38 in LPS-activated RAW264.7 macrophages, while simultaneously upregulating Erg-2, an M2-specific transcription factor (Yu et al., 2019). Similar effects have been demonstrated in osteoarthritis models, where liraglutide facilitates macrophage polarization toward the anti-inflammatory M2 phenotype by downregulating M1-associated markers such as Mcp-1 and Cd38, while upregulating M2-related genes including Erg-2, reducing the secretion of pro-inflammatory cytokines and mitigates inflammatory responses (Meurot et al., 2022). Furthermore, in periodontal disease, liraglutide was also found to downregulate M1-associated surface markers and cytokines, such as CD11c (Itgax), thereby reducing the proportion of M1 macrophages in gingival tissues and alleviating alveolar bone loss. This further confirms the universality of the role of liraglutide in regulating macrophage polarization across different diseases (Sawada et al., 2020).

#### 2.2.4 Endoplasmic reticulum stress

Endoplasmic reticulum (ER) stress plays a central role in metabolic, vascular, and neuroinflammatory disorders. GLP-1 signaling demonstrates potent endoplasmic reticulum (ER) stress-modulating effects in diverse pathological contexts. In obese (OB/OB) mice, GLP-1 reduces ER stress in adipose tissue, improving insulin sensitivity through unresolved mechanisms (Jiang et al., 2018; Yi et al., 2023). In mouse islets, ER induces a shift in GLP-1R coupling from Gs to Gq proteins. Notably, chemical chaperones (4-PBA, TUDCA) enhance Gq signaling without reversing this switch, while ER stress-associated UPR factors XBP1 and ATF6 selectively amplify Gs signaling. Furthermore, Gs utilization in GLP-1R signaling was enhanced by X-box binding protein 1 (XBP1) and activating transcription factor 6 (ATF6)—two key transcription factors involved in endoplasmic reticulum (ER) stress responses, have been shown to selectively enhance Gs signaling through GLP-1R, whereas Gq signaling remains unaffected (Gao et al., 2024). In vascular dysfunction, Exendin-4 attenuates hyperhomocysteinemia (HHcy)-induced endothelial ER stress via AMPK-dependent upregulation of ERO1α, restoring vascular function (Sawada et al., 2020; Cheng et al., 2021). In a mouse model of diabetic retinal neurodegeneration, GLP-1RAs can also inhibit ER stress. This effect is mediated through modulation of the Trx–ASK1 complex and stimulation of the Erk signaling cascade, ultimately contributing to the attenuation of oxidative stress (Liu et al., 2020a).

Additional evidence also indicates that activation of the GLP-1/GLP-1R signaling pathway is implicated in the pathogenesis of sepsis-associated encephalopathy (SAE). Specifically, GLP-1R engagement may alleviate endoplasmic reticulum (ER) stress, suppress microglial overactivation, and attenuate the release of pro-inflammatory cytokines and hippocampal neuronal apoptosis. These effects collectively contribute to improved survival outcomes and cognitive function in cecal ligation and puncture (CLP) mouse models.

#### 2.2.5 Metabolic reprogramming

Metabolic reprogramming refers to the process in which cells, under specific physiological and pathological conditions, systematically adjust and transform their metabolic patterns to adapt to changes in the external environment and to meet their own needs for growth and differentiation, by engaging various bioenergetic routes, such as glucose utilization, lipid metabolism, and amino acid metabolism (Xia et al., 2021; Yang et al., 2023). Increased evidence suggests that metabolic reprogramming is closely related to the progression of diseases and drug resistance. Its diversity and vulnerability make the involved pathway proteins potential therapeutic targets (Horn and Tacke, 2024; Bertels et al., 2024). Experimental studies demonstrate that GLP-1–based therapies exert significant effects on metabolic remodeling. In mice upon diet-induced obesity (DIO), G49 (GLP-1R and glucagon receptor (GCGR) dual agonists, dualAG) can induce metabolic reprogramming of adipocytes, it can stimulate lipolysis in white adipose tissue, leading to increased circulating free fatty acids (FFAs). These FFAs are then transported to the liver, where they undergo FAO, resulting in increased ketogenesis. The increase of FFAs can also activate the liver to produce fibroblast growth factor 21 (FGF21), GLP-1RAs can also activate brown adipose tissue (BAT) by up-regulating uncoupling protein 1 (UCP1) expression, thereby elevating energy expenditure and promoting weight reduction, which further enhances metabolic reprogramming (Valdecantos et al., 2024). Beyond preclinical evidence, clinical data underscore the therapeutic relevance of GLP-1RAs. In a large retrospective cohort study, GLP-1RAs significantly improve metabolic outcomes in individuals with MASLD, it can reduce major adverse cardiovascular events, all-cause mortality, and hospitalizations due to heart failure among individuals diagnosed with type 2 diabetes (Havranek et al., 2025), thereby offering promising strategies for the management of obesity, diabetes, and related cardiometabolic disorders.

### 2.3 Crosstalk between GLP-1 and and the gut microbiome

#### 2.3.1 GLP-1 modulates the gut microbiota

Emerging evidence indicates a bidirectional regulatory relationship between GLP-1RAs and the gut microbiota (Zhang et al., 2018; Zhao et al., 2018). In patients with type 2 diabetes mellitus, metagenomic analyses reveal that GLP-1RAs induce structural and functional remodeling of gut microbial communities, ameliorating dysbiosis and improving host metabolic homeostasis (Shang et al., 2021). Similarly, in male rats with type II diabetes, administration of liraglutide selectively promoted the enrichment of short-chain fatty acid (SCFA)-producing taxa, including *Bacteroides*, Lachnospiraceae, and other beneficial probiotic species. Moreover, diabetic rats typically exhibit a markedly elevated Firmicutes-to-Bacteroidetes (F/B) ratio compared to healthy controls, which is reversed following liraglutide treatment (Zhang et al., 2018). In a diabetic kidney disease (DKD) model, liraglutide was demonstrated to enhance renal outcomes through the modulation of gut microbiota composition and the elevation of circulating levels of L-5-Oxoproline (5-OP). This metabolite was discovered to mitigate ectopic lipid deposition in renal tubular cells, indicating the presence of a gut microbiota–5-OP–kidney axis (potentially mediated by Clostridium–5-OP–ELD), which may serve as a novel intervention target (Steven et al., 2017; Yi et al., 2024).

Besides diabetes, GLP - 1RAs have exhibited gut microbiota - modifying effects in other metabolic disorders, especially some bacteria associated with glycolipid metabolism and intestinal inflammation (Liu et al., 2020b; Madsen et al., 2019). In patients with NAFLD, liraglutide enriched the abundance of *Firmicutes*, *Bacteroidetes*, and *Actinomycetes*, accompanied by a reduction in *Proteobacteria*, which contains many pathogens (Ying et al., 2023). In mice suffering from non - alcoholic fatty liver disease (NAFLD), liraglutide significantly elevated the abundance of *Akkermansia*, *Romboutsia*, and specific members of *Bacteroidales, while* reducing that of *Klebsiella*, *Anaerotruncus*, *Bacteroides*, and several taxa within the *Lachnospiraceae* and *Ruminococcaceae* families (Kim et al., 2022). Similar microbial shifts have been reported in obese mice, wherein liraglutide decreased species within *Firmicutes* (e.g., *Lachnospiraceae*, *Clostridiales*) and increased beneficial taxa like *Verrucomicrobia* and *Oscillospiraceae* (Madsen et al., 2019). Semaglutide also exerted protective effects by reversing high-fat diet (HFD)-induced gut dysbiosis. The treatment increased the abundance of beneficial genera such as *Lachnospiraceae*, *Ruminococcus*, and *Akkermansia*. These taxa were inversely correlated with proinflammatory cytokines including IL-1β, IL-6, and TNF-α (Wang et al., 2016; Duan et al., 2024). At the same time, semaglutide significantly promotes the growth of *Bacteroides acidifaciens* and *Blautia coccoides*, which may contribute to the production of acetate (Da Silva et al., 2024).

Furthermore, Kato et al. demonstrated that liraglutide modulates the composition of obesity-associated gut microbiota, such as *Escherichia coli*, markedly restructuring the microbial community and reshaping bacterial taxa linked to body weight regulation, thereby contributing to weight reduction (Kato et al., 2021).

Nevertheless, there are also some contradictory results. In a 3-month randomized controlled study of type 2 diabetes, it was found that the combination of liraglutide and sitagliptin had no effect on the composition of intestinal flora, had no effect on the alpha and beta diversity, but liraglutide resulted in an elevation of deoxycholic acid (DCA) levels, indicating potential modulation of bile acid metabolism (Smits et al., 2021). In addition, the drug combination also had no effect on the intestinal microbiome in elderly patients with T2DM, but there was an increase in the percentage of bacteria of the genus Alistipes. Though it was not statistically significant. This result may be related to insufficient sample size (Rizza et al., 2023). In this study, the subjects’ diets were not standardized and the treatment with liraglutide may have altered food intake, leading to different results.

In summary, current evidence indicates that GLP-1RAs modulate gut microbiota in ways that may underlie their metabolic and organ-protective effects, although heterogeneity between models and trials remains. These findings are systematically collated in Table 1, which provides an overview of microbial taxa altered by GLP-1RAs (Table 1).

**TABLE 1:** Gut microbiota alterations induced by GLP-1RAs.

GLP-1RAs	Altered bacterial taxa (↑ increase/↓ Decrease)	References
Liraglutide	↑ SCFA-producing taxa (e.g., *Bacteroides*, *Lachnospiraceae*) ↓ Firmicutes-to-Bacteroidetes (F/B) ratio ↑ *Akkermansia*, *Romboutsia*, specific members of *Bacteroidales* ↓ *Klebsiella*, *Anaerotruncus*, *Bacteroides*, *Lachnospiraceae*, *Ruminococcaceae* ↑ *Firmicutes*, *Bacteroidetes*, *Actinomycetes* ↓ *Proteobacteria* ↑ *Escherichia coli* (restructuring effect) ↑ *Alistipes* (not statistically significant)	Zhang et al., 2018; Zhao et al., 2018; Shang et al., 2021; Yi et al., 2024; Liu et al., 2020; Ying et al., 2023; Kim et al., 2022; Kato et al., 2021; Rizza et al., 2023
Semaglutide	↑ *Lachnospiraceae*, *Ruminococcus*, *Akkermansia* ↑ *Bacteroides acidifaciens*, *Blautia coccoides* ↓ Microbial dysbiosis induced by high-fat diet (overall reversal)	Duan et al., 2024; Da Silva et al., 2024
Liraglutide + Sitagliptin	No significant change in α- and β-diversity ↑ Deoxycholic acid (DCA) ↑ ***Alistipes*** (not statistically significant)	Smits et al., 2021; Rizza et al., 2023

#### 2.3.2 Gut microbiota and its metabolites modulate GLP-1 secretion

Emerging evidence highlights a bidirectional regulatory interplay between the gut microbiota and GLP-1, whereby microbial composition and metabolites exert profound influences on GLP-1 synthesis, secretion, and signaling. This interaction extends beyond glucose regulation and may provide novel therapeutic targets for metabolic and inflammatory diseases.

In animal models, depletion of gut microbiota—whether through germ-free status or antibiotic treatment—has been shown to disrupt postprandial GLP-1 secretion, underscoring the critical role of microbial communities in enteroendocrine function (Guo et al., 2013; Wichmann et al., 2013; Grasset et al., 2017). In 2023, Liu et al. demonstrated, using antibiotic-treated (ABX) and germ-free (GF) mouse models, that microbial depletion in the ileum abolished the postprandial GLP-1 response following oral gavage of olive oil. This effect was particularly pronounced in the ileal region of the circulation. Additionally, the postprandial elevation of bile acid levels—which are known to trigger GLP-1 secretion—was markedly suppressed. Notably, restoration of GLP-1 secretion was achieved by fecal microbiota transplantation (FMT) or supplementation with ω-muricholic acid (ωMCA) and hyodeoxycholic acid (HCA) (Wang et al., 2023a). Treatment of SPF mice with vancomycin disrupts gut microbiota balance, characterized by reduced microbial diversity and altered bacterial abundance patterns. Notably, *Lactobacillus* abundance shows significant elevation. These microbial changes significantly enhance GLP-1 activity (Xu et al., 2019). Similarly, *in vitro* experiments, approximately 45 bacterial phylotypes were identified to enhance GLP-1 secretion through exposure of NCI H716 L-cells to their cell-free culture supernatants (Tomaro-Duchesneau et al., 2020), co-incubation of Caco-2 cells with *Enterococcus faecalis* or *Mitsuokella multacida* supernatants led to a significant reduction in GLP-1 mRNA expression (Fredborg et al., 2012). In addition, *E. faecalis* supernatants were capable of disrupting the colonic epithelial monolayer and cleaving GLP-1 in a gelE-dependent manner (Levalley et al., 2020).

A novel protein, P9, derived from *Akkermansia muciniphila*, has been shown to stimulate GLP-1 secretion and enhance glucose homeostasis in mice subjected to a high-fat diet (HFD) (Cani and Knauf, 2021). Lai et al. also discovered that some specific gut microbiota such as *L. reuteri* and *B. thetaiotaomicron* regulates exercise activity by reducing the levels of GLP-1 through vagus nerve-dependent GLP-1 signaling pathway (Lai et al., 2024). However, there are also some studies showed that some specific bacterial species can inhibit the section of endogenous GLP-1. For example, *Clostridium butyricum* (*Cb*) treatment reversed MPTP-induced intestinal microbiota dysregulation and decreased GLP-1, GPR41/43 and brain GLP-1 receptor levels in mice (Yoon et al., 2021; Sun et al., 2021).

Microbial metabolites—postbiotics—also serve as potent regulators of GLP-1 signaling (Greiner and Backhed, 2016). Indole and short-chain fatty acids (SCFAs) such as acetate, propionate, and butyrate have been shown to promote GLP-1 secretion via GPR41/43-mediated pathways (Chimerel et al., 2014; Kaji et al., 2011; Tolhurst et al., 2012). Notably, SCFA-mediated GLP-1 release was abolished in *Gpr43*-deficient mice, confirming receptor dependency (Psichas et al., 2015). Study found that increasing endogenous and exogenous intestinal SCFAs can stimulate intestinal endocrine cells to secrete GLP-1 and PYY by binding to GPR41 and GPR43 (Canfora et al., 2019). Additional findings highlight that dietary compounds like sulforaphane (SFN) may potentiate SCFA signaling to augment GLP-1 release while concurrently attenuating intestinal inflammation (Tian et al., 2024). Beyond these classical metabolites, elevated L-tryptophan levels can also promote GLP-1 secretion in mouse intestinal L cells and contribute to β-cell regeneration in db/db mice. Mechanistically, L-tryptophan upregulated the expression of GLP-1–related genes (e.g., *Gcg* and *Pcsk1*) in intestinal L cells, further promoting GLP-1 production (Jiang et al., 2024).

In contrast, some postbiotics may suppress GLP-1 signaling. For instance, the metabolite hydrogen sulfide (H_2_S), which was produced by *Desulfovibrio* has been shown to impair GLP-1 expression, thereby disrupting host glucose metabolism (Qi et al., 2024). This discovery provides new possibilities for the treatment of metabolic syndrome (MetS).

Taken together, these findings demonstrate that gut microbiota and their metabolites exert dual and context-dependent effects on GLP-1 secretion and signaling. While beneficial microbes and postbiotics enhance GLP-1 activity to support metabolic homeostasis, pathogenic species and harmful metabolites may inhibit this pathway, contributing to disease progression. This duality underscores the therapeutic potential of targeting microbiota–GLP-1 interactions in metabolic disorders.

#### 2.3.3 GLP-1, inflammation and the gut microbiome

In view of the above, increasing evidence indicates a complex and reciprocal relationship among glucagon-like peptide-1 (GLP-1), gut microbiota, and inflammation. An imbalance in the gut microbial community can trigger inflammatory responses and modulate GLP-1 secretion, while GLP-1 itself may influence both immune signaling and microbial composition.

Lipopolysaccharide (LPS), a prototypical bacterial endotoxin, has been identified as a key mediator linking microbial dysbiosis to GLP-1 activation. Both animal and human studies demonstrate that LPS stimulate GLP-1 release via Toll-like receptors (TLRs). For example, following the induction of intestinal ischemia in humans, a rapid increase in GLP-1 secretion was observed, suggesting a link between endogenous LPS translocation and GLP-1 activation under conditions of barrier dysfunction (Cani et al., 2007; Lebrun et al., 2017). In parallel, aberrant gut microbiota has been associated with reduced epithelial integrity and increased LPS leakage into systemic circulation, thereby initiating chronic inflammation (Lebrun et al., 2017). Proinflammatory mediators such as interleukin-6 (IL-6), interleukin-1 (IL-1), and LPS have also been reported to directly stimulate GLP-1 secretion from intestinal endocrine L cells. In metabolic disorders such as type 2 diabetes (T2D), microbial dysbiosis is characterized by an increase in Gram-negative Enterobacteriaceae and a reduction in short-chain fatty acid (SCFA)-producing bacteria such as Bifidobacterium, resulting in elevated circulating LPS levels and metabolic endotoxemia (Kahles et al., 2014; Nguyen et al., 2014). Disturbances in the gut microbiota promote endotoxemia and insulin resistance. In individuals with Type 2 Diabetes (T2D), there is an observed increase in Gram-negative *Enterobacteriaceae* and a concurrent decrease in acetic acid-producing bacteria, such as *Bifidobacteria*. This shift in microbial composition leads to an elevation of Lipopolysaccharide (LPS).

Conversely, GLP-1 receptor agonists (GLP-1RAs) have demonstrated beneficial immunomodulatory effects. As shown by Sun et al., treatment with GLP-1RAs reshaped gut microbial composition by increasing the abundance of beneficial genera such as *Lactobacillus* and Bifidobacterium. This microbial shift enhanced the activity of group 3 innate lymphoid cells (ILC3s), thereby promoting anti-inflammatory cytokines IL-22 and IL-10 (Sun et al., 2024).

**FIGURE 2:**
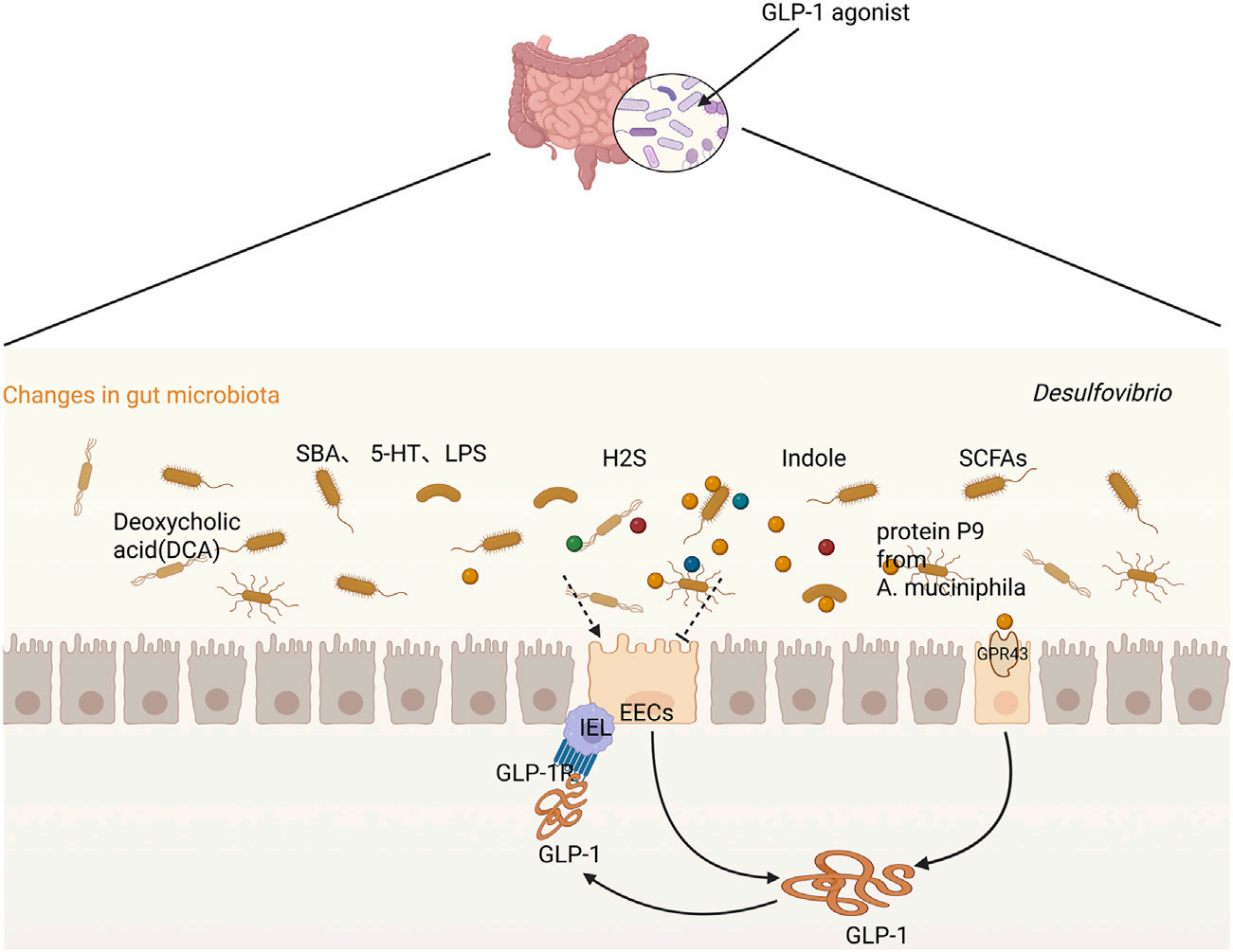
Crosstalk between GLP-1 and the gut microbiome/metabolites.

Taken together, GLP-1, inflammation, and the gut microbiota form a tightly interlinked feedback loop. The microbiota modulates GLP-1 secretion through microbial metabolites; GLP-1, in turn, influences both immune responses and microbial homeostasis; and inflammation both results from and contributes to dysbiosis and altered GLP-1 levels. This tripartite interaction plays a central role in the pathogenesis of various diseases, particularly metabolic disorders such as type 2 diabetes and obesity (Figure 2).

## 3 Clinical applications of GLP-1RAs: comprehensive protection through multi-disease risk reduction

### 3.1 Cardiovascular protection

GLP-1RAs confer substantial cardiovascular benefits that extend beyond glycemic control, primarily by reducing the incidence of major adverse cardiovascular events (MACE). In landmark trials such as LEADER and SUSTAIN-6, liraglutide significantly lowered the risk of cardiovascular death, non-fatal myocardial infarction, and stroke by 13% compared to placebo (Marso et al., 2016a). Similarly, semaglutide was shown to reduce the incidence of nonfatal stroke by 26% (Marso et al., 2016b). These benefits are thought to arise from multiple mechanisms, including improvements in endothelial function, inhibition of vascular inflammation, and enhanced myocardial metabolism via cAMP/PKA and AMPK pathways. A recent meta-analysis involving 32,884 patients from 19 randomized controlled trials further confirmed a 23% reduction in all-cause mortality associated with GLP-1RAs use (OR = 0.77) (Rivera et al., 2024). More recently, the SUMMIT trial demonstrated that tirzepatide reduced the composite risk of cardiovascular death or worsening heart failure by 38% (HR = 0.62, 95% CI: 0.41–0.95) in patients with heart failure with preserved ejection fraction (HFpEF) and a BMI ≥ 30 kg/m^2^, 32% of whom also had type 2 diabetes. This is the first pharmacological agent to show prognostic benefit in this population, offering therapeutic potential beyond symptomatic control. In contrast to traditional diuretics that primarily offer symptomatic management, this novel therapeutic agent demonstrates dual breakthroughs in both pathophysiological intervention and prognostic improvement.

These consistent benefits have been rapidly translated into practice. Current ESC guidelines assign a Class I recommendation to GLP-1RAs for patients with type 2 diabetes and established cardiovascular disease. And in March 2024, the U.S. FDA approved a new indication for semaglutide (Wegovy) to reduce the risk of cardiovascular death, myocardial infarction, and stroke in adults with CVD and either obesity or overweight (Lincoff et al., 2023).

### 3.2 Neurological and psychosocial protection

GLP-1RAs have demonstrated growing potential in neurological and psychosocial disorders. In Alzheimer’s disease (AD), both preclinical and clinical studies have demonstrated consistent benefits. Semaglutide has been shown to reduce β-amyloid deposition, improved spatial memory, and enhanced cerebral glucose metabolism (Zhang et al., 2024). In a UK clinical trial of 204 AD patients, liraglutide slowed regional brain volume loss by nearly 50%, as assessed by MRI (Femminella et al., 2019)-scale retrospective analyses further support these findings. Large-scale real-world analyses further reinforced these findings: a U.S. national electronic health record study of over one million individuals with type 2 diabetes revealed that semaglutide use was associated with a 70% reduced risk of AD compared to insulin (HR = 0.33, 95% CI: 0.21–0.51), and a 40% reduction compared to other GLP-1 drugs (HR = 0.59, 95% CI: 0.37–0.95) (Wang et al., 2024). Similar risk reductions were observed in cohorts from Oxford and the U.S. Department of Veterans. The cohort study from the University of Oxford (n ≈ 100,000) showed a 48% lower risk of dementia among semaglutide users compared to those treated with sitagliptin. In the U.S. Department of Veterans Affairs database found that GLP-1RA therapy reduced the risk of dementia and AD by 8% and 12%, respectively (Xie et al., 2025).

Evidence is also accumulating in Parkinson’s disease, where GLP-1RAs have shown neuroprotective actions. In experimental models, they preserved dopaminergic neurons and improved motor performance via mitochondrial and endoplasmic reticulum stress pathways (Sedky and Magdy, 2021). A clinical trial using lixisenatide in early Parkinson’s patients demonstrated slowed progression of motor symptoms: the treated group had stable MDS-UPDRS Part III scores (Δ = −0.04), while the placebo group showed worsening disability (Δ = +3.04) (Malhotra et al., 2024).

Evidence also suggests antidepressant effects of GLP-1RAs. In diabetic mice, semaglutide alleviated anxiety- and depression-like behaviors and improved cognitive performance via modulation of the microbiota-gut-brain axis (de Paiva et al., 2024). A meta-analysis of five RCTs involving 2,000 participants indicated that GLP-1RA-treated adults had significantly lower depression scale scores than controls. Furthermore, real-world data from Epic Research suggested that users of tirzepatide, semaglutide, dulaglutide, or exenatide had a lower risk of depression than non-users, although this study is pending peer review (Chen et al., 2024).

In summary, accumulating evidence supports the potential of GLP-1RAs in treating neurodegenerative and psychosocial disorders. These benefits may be attributed to their anti-inflammatory, antioxidant, and neuroprotective properties, as well as improvements in brain energy metabolism (De Giorgi et al., 2025).

### 3.3 Renal protection

Kim et al. (2013) first identified that GLP-1 receptor agonists (GLP-1RAs) enhance renal perfusion, a mechanism mediated by increased atrial natriuretic peptide (ANP) secretion. Subsequent studies in knockout mice confirmed that ANP signaling plays a central role in GLP-1RA-induced renal protection (Kim et al., 2013). In STZ-induced diabetic rats, Yin et al. (2019) further demonstrated that recombinant human GLP-1 (rhGLP-1) suppresses epithelial apoptosis and mitigates tubulointerstitial fibrosis via the PKA/PKC signaling pathway (Yin et al., 2019).

These preclinical insights have been validated in large-scale clinical trials.The FLOW trial (n = 3,533) showed that once-weekly subcutaneous administration of semaglutide (1.0 mg) significantly reduced the risk of a renal composite endpoint—including ≥50% decline in eGFR, renal failure, or cardiovascular death—by 24% in patients with T2DM and chronic kidney disease (CKD) (HR = 0.76, 95% CI: 0.66–0.88; p < 0.001) (Perkovic et al., 2024; Rossing et al., 2023). A meta-analysis of 12 randomized controlled trials (n = 17,996) further confirmed these renoprotective effects, with agents structurally closer to endogenous human GLP-1, such as semaglutide, showing the strongest benefit (RR = 0.78, 95% CI: 0.71–0.85) (Chen et al., 2025). Based on these results, the U.S. FDA approved semaglutide (Ozempic) on 28 January 2025 for reducing the risk of kidney disease progression, kidney failure (end-stage renal disease), and cardiovascular death in adults with type 2 diabetes and chronic kidney disease. The approval of Simiglutide for CKD indication, representing a significant advancement for patients with T2DM combined with CKD.

### 3.4 Hepatic protection

In clinical practice, semaglutide has demonstrated notable efficacy in resolving NASH without aggravating hepatic fibrosis. A key double-blind, placebo-controlled phase II study conducted over 72 weeks recruited 320 individuals with histologically confirmed NASH and liver fibrosis classified as stage F1 to F3. The proportion of patients achieving NASH resolution in the absence of fibrosis progression was 40%, 36%, and 59% for the 0.1, 0.2, and 0.4 mg semaglutide arms, respectively, whereas the corresponding rate in the placebo arm was 17% (Newsome et al., 2021). Semaglutide was the first GLP-1RA to meet the primary endpoint in a phase II NASH trial. Additionally, tirzepatide—a dual GIP/GLP-1 receptor agonist—demonstrated substantial efficacy in patients with metabolic dysfunction-associated steatohepatitis (MASH) and liver fibrosis. In a separate study involving 190 patients receiving 52 weeks of treatment, remission rates were 36.8%, 45.6%, and 52.0% for the 5, 10, and 15 mg tirzepatide groups, respectively, compared to 18.2% in the placebo group (Loomba et al., 2024). Finally, GLP-1RAs have also been associated with improvements in biochemical parameters, imaging markers, and histological features of fatty liver and fibrosis in patients with nonalcoholic fatty liver disease (NAFLD) (Nevola et al., 2023).

Notably, based on the results from part 1 of the ESSENCE trial, on 15 August 2025, the FDA approved semaglutide 2.4 mg for the treatment of adults with MASH with moderate to advanced liver fibrosis (consistent with stages F2 to F3 fibrosis). At week 72, the clinical data showed that 36.8% of patients receiving semaglutide achieved fibrosis improvement without worsening of NASH and 62.9% achieved NASH resolution without worsening of fibrosis, compared with 22.4% and 34.3%, respectively, in the placebo group (Sanyal et al., 2025). This approval provides a new therapeutic option for MASH, capable of halting progression and reversing established hepatic injury.

### 3.5 Respiratory protection

In December 2024, tirzepatide received FDA approval for the treatment of moderate-to-severe obstructive sleep apnea (OSA) in obese adults (Beccuti et al., 2024). Furthermore, evidence from a large population-based cohort study demonstrated that, compared with sulfonylureas, GLP-1RAs were associated with a 30% reduction in the risk of severe exacerbations among patients with chronic obstructive pulmonary disease (COPD). In the context of asthma, GLP-1RAs have been shown to induce pulmonary vasodilation and stimulate phosphatidylcholine secretion via activation of the cAMP-dependent protein kinase A (PKA) signaling pathway, thereby enhancing respiratory function (Yu et al., 2023).

Preclinical studies using COPD mouse models revealed that GLP-1RA therapy significantly improved lung function and reduced both morbidity and mortality. Clinical investigations further confirmed that GLP-1RA treatment led to significant improvements in key pulmonary function parameters, including forced expiratory volume in one second (FEV_1_), forced vital capacity (FVC), and mid-expiratory flow rates (MEF_75_ and MEF_50_). Additionally, treatment with GLP-1RAs was found to upregulate the expression of pulmonary surfactant proteins A and B, contributing to improved respiratory physiology (Wang et al., 2023b).

Collectively, these data position GLP-1RAs as promising agents for the management of respiratory diseases, offering both symptomatic relief and potential disease-modifying effects.

### 3.6 Reproductive protection

Glucagon-like peptide-1 (GLP-1) has emerged as an important regulator of reproductive function through direct and indirect effects on the hypothalamic–pituitary–gonadal (HPG) axis. Preclinical studies suggest that GLP-1 and its analogs may exert direct effects on the hypothalamic–pituitary–gonadal (HPG) axis, thereby modulating reproductive function. In rodent models, both GLP-1 and exendin-4 appear to influence reproductive efficiency via interaction with the hypothalamic kisspeptin (Kiss-1) system (Outeirino-Iglesias et al., 2015). Notably, improvements in reproductive dysfunction have been observed in several animal models of polycystic ovary syndrome (PCOS), including enhanced follicular development, normalized estrous cycles, and reversal of polycystic ovarian morphology (Singh et al., 2019; Tao et al., 2019). In a postweaning androgenization (PWA) mouse model, treatment with a GLP-1/GIP/glucagon triagonist significantly reduced ovarian weight and serum luteinizing hormone (LH) levels. Similar reductions in serum LH were reported with GLP-1/GIP administration, while GLP-1/E treatment restored estrous cyclicity and increased the number of corpora lutea across multiple generations (Sanchez-Garrido et al., 2024). Interestingly, agmatine, a metabolite produced by the gut bacterium *Bacteroides vulgatus*—was shown to impair ovarian function by inhibiting GLP-1 secretion through activation of the farnesoid X receptor (FXR) (Yun et al., 2024). A systematic review and meta-analysis of 11 randomized controlled trials involving 840 women with PCOS concluded that GLP-1RAs significantly improve natural conception rates (RR: 1.72, 95% CI: 1.22–2.43; p = 0.002; I^2^ = 0%), menstrual regularity, insulin sensitivity, and multiple anthropometric and hormonal parameters (Zhou et al., 2023). GLP-1RAs may provide therapeutic opportunities in conditions such as PCOS and obesity-related infertility.

In parallel, emerging data from male reproductive studies suggest that GLP-1RAs may positively influence the hypothalamic-pituitary–testicular axis, improve testicular function, and enhance spermatogenesis, particularly in obese men. However, additional studies are warranted to validate these findings (Varnum et al., 2023).

### 3.7 GLP-1RAs and obesity-associated cancers

Obesity is a well-recognized risk factor for a range of malignancies. In 2016, the International Agency for Research on Cancer identified 13 obesity-associated cancers (OACs) with increased incidence and poorer prognosis among people with excess body weight. Owing to its pronounced effects on body-weight reduction, glycemic regulation, and attenuation of systemic inflammation, GLP-1RAs have attracted growing interest as potential modulators of cancer-relevant biological pathways. A synthesis of preclinical and population-level evidence now indicates that these agents may influence the development and progression of several OACs, particularly hepatocellular carcinoma (HCC), pancreatic cancer, and colorectal cancer (CRC).

In Colorectal cancer, Ma et al. (2023) reported that exposure to high glucose markedly promoted proliferation, migration, and invasion in colorectal cancer cell lines (SW480, HCT116), and accelerated tumor growth in a subcutaneous xenograft model. Mechanistically, high-glucose conditions induced robust upregulation of BMP4 and increased phosphorylation of its downstream effectors Smad1/5/9, driving tumorigenic signaling. Concurrently, high glucose suppressed the tumor suppressor TIF1γ, further amplifying Smad-mediated transcription and elevating the expression of pro-oncogenic genes, including the epithelial–mesenchymal transition markers N-cadherin and vimentin, thereby collectively fostering CRC progression. Treatment with GLP-1 receptor agonists partially reversed these changes by lowering BMP4 expression, suppressing tumor growth, and inducing apoptosis (Ma et al., 2023).

In preclinical models of pancreatic cancer, Zhao et al. found that GLP-1R activation reduced tumor growth and metastatic potential by inhibiting ERK1/2 and AKT phosphorylation, lowering MMP-2 and MMP-9 expression, and increasing E-cadherin. These effects limited EMT and invasive behavior (Zhao et al., 2014). Findings from a large multicenter analysis of more than 1.5 million adults with type 2 diabetes in six national health databases were in line with these results. The study found no increase in pancreatic cancer risk with GLP-1RA use, and several sensitivity analyses suggested a modest, non-significant reduction in incidence (Wang et al., 2025).

Hepatocellular carcinoma (HCC) often develops in the context of obesity-related liver disease, including nonalcoholic steatohepatitis (NASH). In murine models, GLP-1RAs have been shown to reduce the incidence of HCC associated with NASH (Kojima et al., 2020). In hepatocellular carcinoma models induced by DEN combined with either high-fat/high-carbohydrate or low-fat/low-carbohydrate diets, lean m/db mice, and MCD- or CDE-induced liver injury—as well as in human models, exenatide activation of the GLP-1R–cAMP–PKA–EGFR–STAT3 pathway suppressed oncogenic signaling, limited tumor cell growth, and enhanced apoptosis, resulting in a marked reduction in tumor progression (Zhou et al., 2017). Consistent with these biological effects, data from a large population-based cohort in Taiwan showed that initiation of GLP-1RAs, compared with insulin, was associated with a significantly lower incidence of HCC (HR = 0.47, 95% CI: 0.36–0.61) (Yang et al., 2024).

**FIGURE 3:**
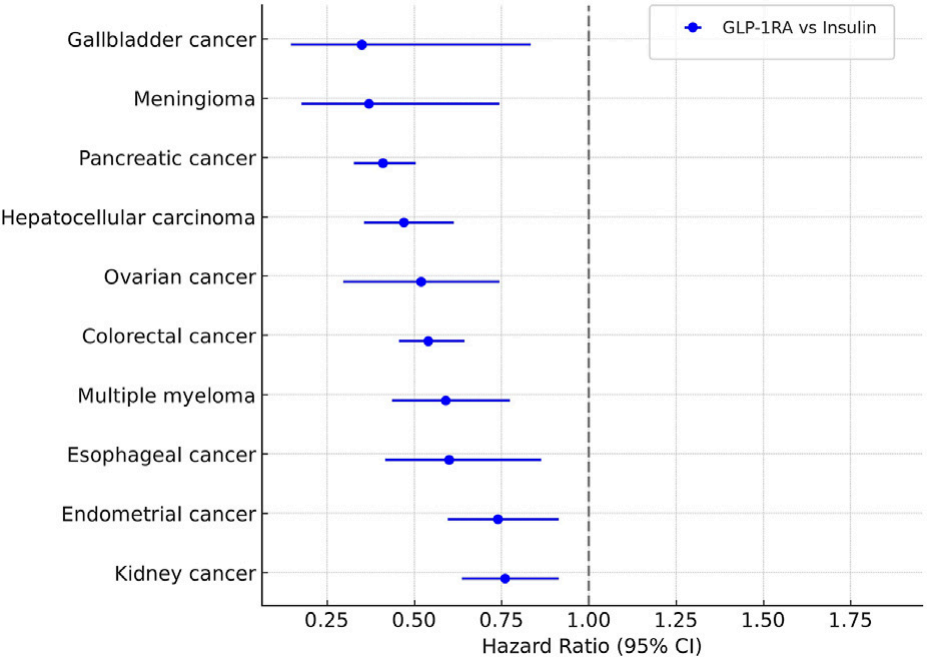
Association between GLP-lRAs use and cancer risk.

Beyond these individual cancers, the cohort analysis showed significantly lower rates of 10 OACs in GLP-1RA users compared with those on insulin. These included gallbladder cancer (HR = 0.35), meningioma (HR = 0.37), pancreatic cancer (HR = 0.41), hepatocellular carcinoma (HR = 0.47), ovarian cancer (HR = 0.52), colorectal cancer (HR = 0.54), multiple myeloma (HR = 0.59), esophageal cancer (HR = 0.60), endometrial cancer (HR = 0.74), and kidney cancer (HR = 0.76). Compared with metformin, hazard ratios for colorectal and gallbladder cancers remained below one, but the differences were not statistically significant (Figure 3).

Taken together, these mechanistic data and large-scale epidemiological findings point to a possible protective effect of GLP-1RAs against selected OACs. These results highlight the need to explore GLP-1–based treatments not only for metabolic disease but also as part of broader cancer-prevention strategies. Further translational research and prospective clinical trials are required to clarify causality, determine effect size, and identify high-risk groups most likely to benefit.

### 3.8 GLP-1 in sepsis: Friend or foe?

Sepsis is a life-threatening condition characterized by a dysregulated host response to infection, resulting in tissue injury, organ dysfunction, and potentially death. It remains a leading cause of mortality and morbidity in intensive care units (ICUs) worldwide (Gotts and Matthay, 2016). Current standard treatment involves broad-spectrum antibiotics and intravenous fluids to stabilize hemodynamics and maintain organ perfusion. However, therapeutic efficacy remains limited, and the economic burden is substantial (Yao et al., 2020).

In preclinical models of polymicrobial sepsis, such as the cecal slurry and cecal ligation puncture (CLP) models, long-acting GLP-1 receptor agonists (GLP-1RAs) like semaglutide have demonstrated protective effects. These include reductions in core body temperature, bacterial burden across multiple organs, and systemic and pulmonary inflammatory cytokines. Mechanistically, GLP-1R activation appears to downregulate TNF-α production via α1-adrenergic, δ-opioid, and κ-opioid receptor pathways (Wong et al., 2024). GLP-1RAs have also been shown to attenuate vascular inflammation and oxidative stress, while preserving endothelial barrier integrity and limiting neutrophil infiltration in models of sepsis-associated acute lung injury (ALI) (Helmstadter et al., 2021; Wiberg et al., 2018).

Clinically, elevated levels of endogenous GLP-1 have been observed in patients with sepsis (6.35-fold increase), end-stage renal disease (4.46-fold), and burns (2.96-fold), indicating a common physiological response to critical illness (Lebherz et al., 2017). In sepsis caused by Gram-negative bacteria, overactivation of GLP-1 within the first 24 h has been associated with increased mortality risk, suggesting a complex and potentially adaptive regulatory role (Brakenridge et al., 2019). Elevated GLP-1 levels may reflect a metabolic shift toward aerobic glycolysis in response to stress and may serve as a marker of disease severity.

Despite these observations, exogenous GLP-1RAs appear to exert beneficial effects. In a clinical trial involving 112 patients, administration of the GLP-1 analogue exenatide led to a 21% faster reduction in blood lactate levels within 6 hours compared to placebo (95% CI: 6.0%–33%; p = 0.02) (Wiberg et al., 2018). Additional experimental data support the protective role of GLP-1, including improved outcomes in DPP-4 knockout rats exposed to endotoxins and reduced mortality following GLP-1R activation (Ku et al., 2010). Therefore, the therapeutic relevance of both endogenous incretin hormones and exogenous GLP-1RAs in sepsis warrants further investigation. In patients with critical sepsis, circulating GLP-1 levels are markedly elevated and have been associated with disease severity and clinical outcomes. However, this elevation should not be interpreted as evidence of a detrimental effect. On the contrary, increased GLP-1 levels are more likely to represent an adaptive, protective response to systemic stress and inflammation (Perl et al., 2018; Lebherz et al., 2017).

**FIGURE 4:**
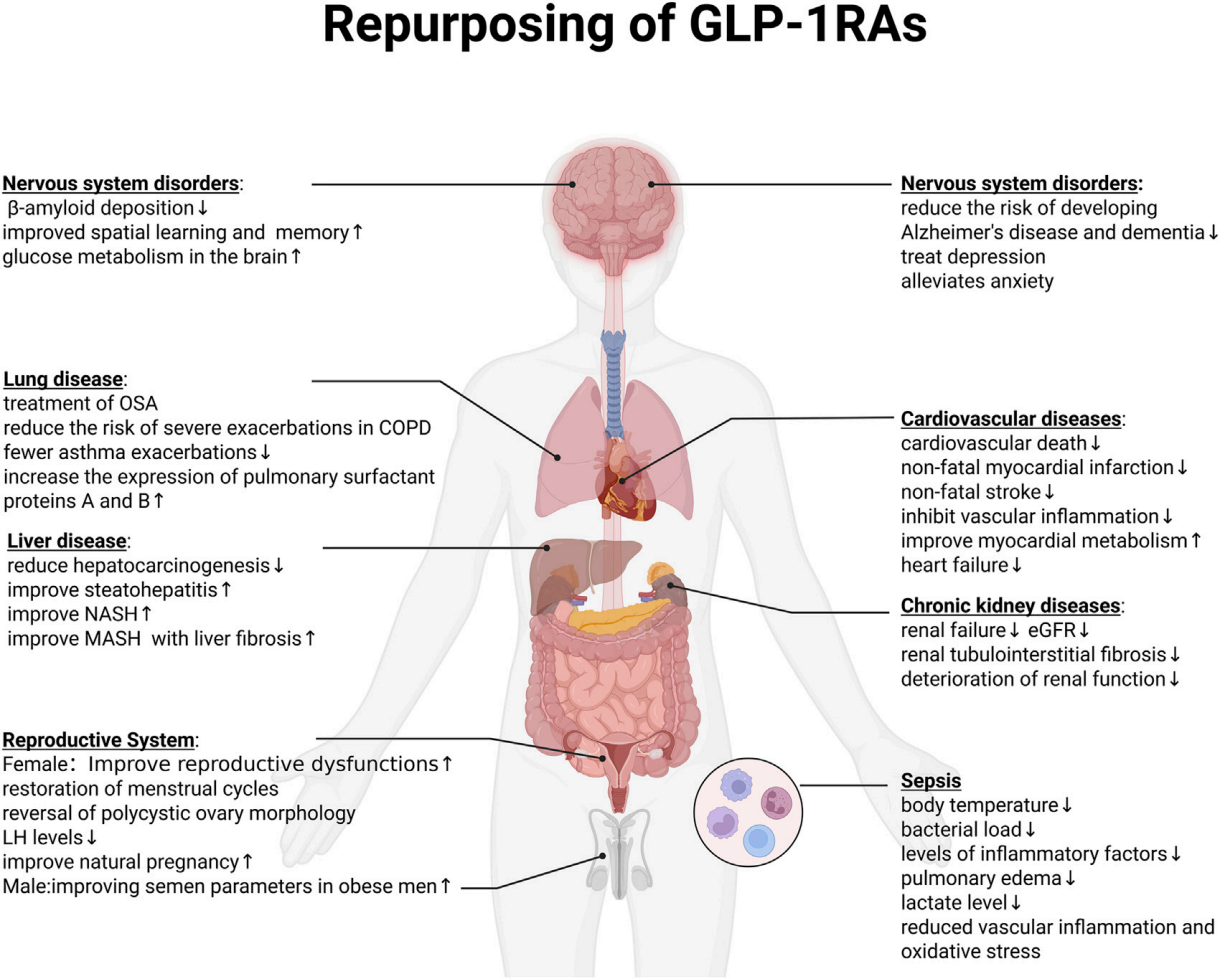
Repurposing of GLP-1RAs.

Taken together, preclinical and clinical studies have demonstrated that GLP-1 receptor agonists exert pleiotropic effects extending beyond glycemic control, influencing multiple organ systems including the nervous, respiratory, hepatic, renal, cardiovascular, reproductive, and immune systems (Figure 4).

## 4 Comparative overview of mainstream GLP-1RAs

Over the past 2 decades, several GLP-1RAs have entered clinical practice, including short-acting exenatide, intermediate-acting liraglutide, and long-acting formulations such as dulaglutide and semaglutide, as well as the newer dual agonist tirzepatide. They share a common therapeutic value: improved glycaemic control, weight reduction, and protection against cardiovascular and renal outcomes. Their clinical utility, however, differs by formulation and pharmacokinetic profile. The long-acting analogues—particularly semaglutide and tirzepatide—achieve the greatest reductions in glycated hemoglobin and body weight, while liraglutide and dulaglutide show moderate but clinically relevant benefits. Exenatide remains effective but is generally less potent in both glycemic and weight outcomes, which has limited its current use (Thomsen et al., 2025).

With regard to adverse effects, gastrointestinal intolerance—especially nausea and vomiting—represents the class-defining limitation. These events are dose-dependent and most pronounced during dose escalation. Semaglutide and tirzepatide are associated with the highest rates of gastrointestinal symptoms, although these tend to attenuate over time and can be mitigated with gradual titration (Rosenstock et al., 2021). Liraglutide and dulaglutide generally show better tolerability but still require careful counseling to maintain adherence (Drucker, 2024). Other safety considerations include rare reports of pancreatitis and the unresolved concern regarding thyroid C-cell tumors, as reflected by boxed warnings (Espinosa et al., 2024; Guo et al., 2024).

Overall, the comparative evidence suggests that long-acting and multi-agonist agents maximize efficacy at the expense of higher gastrointestinal intolerance, whereas older agents provide more modest benefits with relatively favorable tolerability. This balance between efficacy and adverse effects should guide personalized therapeutic selection, particularly in populations with variable comorbidity profiles or adherence challenges (Table 2).

**TABLE 2:** Comparative efficacy and safety profile of mainstream GLP-1 receptor agonists and dual agonists.

Drug/Dose	Duration	HbA1c ↓ (%)	Weight ↓ (%)	MACE HR	Renal endpoint HR	GI adverse events (%)	Pancreatitis risk (%)	MTC contraindication
Exenatide BID	Short	0.8–1.0	2.8	Not proven	Not proven	30–35	0.2	Yes
Lixisenatide QD	Short	0.7–0.9	1.8	1.02	Not proven	20–25	0.1	Yes
Liraglutide 1.8 mg QD	Long	1.1–1.5	3.5–5.4	0.87	0.78	25–30	0.2	Yes
Dulaglutide 4.5 mg QW	Long	1.2–1.8	4.7	0.88	Not proven	20–25	0.1	Yes
Semaglutide 1.0 mg QW	Long	1.5–1.8	6–7	0.74	0.76	30–35	0.3	Yes
Semaglutide 2.4 mg QW	Long	2.0–2.4	12.4	0.80	0.76	40–45	0.3	Yes
Tirzepatide 15 mg QW	Dual-long	2.0–2.5	15.7	0.62	Not reported	40–45	0.2	Yes

## 5 Discussion

GLP-1RAs have emerged as a paradigm-shifting class of agents, with therapeutic benefits that extend well beyond glycemic control in type 2 diabetes. Mounting evidence demonstrates their efficacy in reducing cardiovascular and renal risk, improving hepatic outcomes, and potentially modulating neurological and inflammatory pathways. These pleiotropic effects highlight their potential to transform precision medicine across a spectrum of metabolic and non-metabolic diseases.

Despite these advances, several controversies remain unresolved. One important debate concerns the mechanisms underlying GLP-1RA benefits in non-metabolic diseases. In metabolic dysfunction–associated steatohepatitis (MASH), for example, it remains uncertain whether improvements in hepatic inflammation and fibrosis are exclusively secondary to weight loss and metabolic improvements, or whether direct hepatoprotective mechanisms are involved. Similarly, while cardiovascular protection is consistently observed, the extent to which this arises from direct receptor signaling in cardiomyocytes versus indirect systemic effects remains debated. Safety considerations also warrant continued attention. Gastrointestinal intolerance, particularly nausea and vomiting, may affect adherence despite their tendency to diminish over time (Liu et al., 2022). Concerns about thyroid cancer risk and the potential for sarcopenia or bone fragility in older patients further underscore the need for long-term surveillance (Nunn et al., 2024). Moreover, durable weight management remains challenging. Although GLP-1RAs can reduce body weight by 15%–25% in controlled settings, real-world adherence is limited, and nearly 70% of patients experience weight regain after discontinuation, raising the risk of long-term pharmacological dependence (Jensen et al., 2024).

Looking ahead, the future of GLP-1–based therapy will depend on addressing safety concerns, improving long-term adherence, and expanding clinical indications. Current developmental strategies—including multi-target agonists, ultra–long-acting formulations, and oral delivery systems—reflect these goals. Long-acting formulations aim to enhance bioavailability, reduce adverse events, and prolong therapeutic duration (Kassem et al., 2024). Agents such as MariTide, with a half-life of up to 21 days, enable monthly or potentially longer dosing intervals, achieving substantial and sustained weight loss in clinical trials (Jastreboff et al., 2025). In parallel, multi-target agents offer synergistic metabolic and weight-lowering effects, as exemplified by retatrutide (GLP-1R/GIPR/GCGR agonist) and NA931 (GLP-1R/GIPR/GCGR/IGF1 agonist), both of which demonstrate potent efficacy with acceptable tolerability (Rosenstock et al., 2023; Jastreboff et al., 2023).

Taken together, these findings suggest that GLP-1RAs are poised to reshape the therapeutic landscape by integrating metabolic, cardiovascular, renal, hepatic, and potentially neurological benefits. At the same time, critical questions regarding mechanisms, safety, and sustainability remain to be resolved. Addressing these challenges while capitalizing on innovations in drug design will be essential for optimizing patient outcomes and realizing the full potential of GLP-1–based therapies.
